# Establishing position papers by the WSES

**DOI:** 10.1186/s13017-018-0163-8

**Published:** 2018-01-15

**Authors:** Miklosh Bala, Jeffry Kashuk, Ernest E. Moore, Fausto Catena, Ari Leppaniemi, Luca Ansaloni, Walter Biffl, Federico Coccolini, Andrew Peitzman, Massimo Sartelli, Michael Sugrue, Gustavo P. Fraga, Salomone Di Saverio, Yoram Kluger

**Affiliations:** 1 Trauma and Acute Care Surgery Unit, General Surgery Department, Hadassah Hebrew University Medical Center, Kiriat Hadassah, POB 12000, 91120 Jerusalem, Israel; 2 Tel Aviv University Sackler School of Medicine, Assia Medical Group, Tel Aviv, Israel; 3 Department of Surgery, University of Colorado, Denver Health Medical Center, Denver, USA; 4 Emergency Department, Maggiore University Hospital, Parma, Italy; 5 Abdominal Center, University Hospital Meilahti, Helsinki, Finland; 6 General Surgery I, Papa Giovanni XXIII Hospital, Bergamo, Italy; 7 Scripps Memorial Hospital La Jolla, La Jolla, USA; 8 Department of Surgery, UPMC, University of Pittsburgh School of Medicine, Pittsburgh, PA USA; 9 Macerata Hospital, Ancona University, Macerata Area, Italy; 10 Letterkenny University Hospital and Donegal Clinical Research Academy, Letterkenny, Ireland; 11 Division of Trauma Surgery, Hospital de Clinica, School of Medical Sciences, University of Campinas, Campinas, Brazil; 12 Department of Surgery, Maggiore Hospital, Bologna, Italy; 13 Department of General Surgery, Rambam Health Care Campus, Haifa, Israel

**Keywords:** Position paper, Emergency surgery, Working group, Medical consensus

## Abstract

A position paper (PP) should establish a unified voice in areas where controversy occurs based upon multiple practices and/or therapeutic choices. Typically, a position paper should elucidate the knowledge gap, followed by an evidence-based review of options, leading to an “endorsed position.” A position paper should represent more than the opinion or consensus of the authors but should present current opinions and practices supported by the World Society of Emergency Surgery (WSES). Accordingly, position papers should require the approval of an expert group of WSES and in parallel be presented at an annual meeting prior to submission for publication.

It is important that a unified approach for drafting of position papers be established and endorsed by WSES in order to establish credibility and prevent misunderstandings during a smooth transition to publication.

The purpose of this article is to suggest a uniform process for the development of WSES guidelines.

## Background

By definition, a position paper (PP) is a written statement from an organization that discusses a contemporary clinical problem and suggests an established and agreed upon approach to this problem by the organization. The other term is a “medical consensus.” According to the Council of Europe, “medical consensus” is a statement on a particular aspect of medical knowledge that generally is evidence-based, state-of-the-art knowledge by a representative group of experts in that area [1]. Its main objective is to recommend to colleagues the best possible and acceptable way to address an issue, and includes diagnosis, management, and operative treatment. PP fuses new information, largely from recent or ongoing research that may have implications for re-evaluation of routine medical practices.

The primary difference between a PP and a clinical practice guideline is that PP synthesizes newly available information and reinforces best medical practices but does not give detailed algorithms or guidelines for practice. Additionally, it is much easier to respond to patient needs with a PP compared with clinical practice guidelines [2]. Thus, consensus statements should provide generalized and not specific algorithms, i.e., PP statements should be independent from regional expertise, technology, and local practice.

Recently, several of the co-authors carried a PP concept from inception, development, presentation at the annual World Society of Emergency Surgery (WSES) meeting, and ultimately publication [3]. Based upon comments received on this process, the current article was formulated in order to serve as an example of how to generate a PP from the WSES.

The aim of this overview is to develop common practical steps in planning, preparing, and publishing a PP endorsed by the WSES.

## Format for position papers

### Title

The title should include key information to attract the reader’s interest with a brief quote or detail. It should be connected to the WSES and express the ownership and endorsement of the society.

### Authorship

Ethical guidelines for authorship have been published elsewhere [4, 5] and are included in the “information for authors” section in all peer review journals. Of note, authors should have conscientiously contributed to the formation and construct of the manuscript. The current recommendations of the WSES journal state very clearly: “We recommend adhering to the guidelines for authorship that are applicable in your research field or to the International Committee of Medical Journal Editors (ICMJE) guidelines.” [6] Given the depth and breadth of a PP, there will generally be several authors working together to formulate the plans for proceeding on the process leading to publication. It is critically important that the assignment of author responsibilities be delineated clearly early in the project. Since a PP will generally involve extensive collaboration of many contributors, the best way to ensure delineation of responsibility is to divide topics into various sections, with establishment of a section author who will ultimately be responsible for writing the particular section where their work has focused. These authors will generally be responsible for presentations at the annual meeting relating to their particular section of the PP. The first three authors are responsible for collating all of the section information, presentation comments, and section manuscripts, and organizing the material in a cogent manner for drafting the initial manuscript. The order of priority of the first three authors should be determined ahead, but the first two authors are usually most intimately involved in the initial idea for the manuscript and the third author can serve as an additional advisor during the drafting process. In the example presented [2], the first three authors served in this role and, in addition, served as moderators as well as presenters during the session at the annual meeting when the topic was presented. Other authors who contribute directly to the drafting of the manuscript should be included as co-authors, and the senior author position may be reserved for a senior member who serves as a reviewer and advisor to all co-authors. This plan, as outlined, serves to ethically include all individuals who actively participate in the work; however, it should be emphasized that others who have little to no input should not be included.

### Abstract

The abstract contains a synopsis of the key elements of the paper. The knowledge gap should be defined. The intent is to convey to the reader why the presentation is significant. The abstract should be written in the future tense, as the reader has not yet read the paper. Hence, it is suggesting what the reader may learn as opposed to what he may already know.

### Introduction

The introduction should clearly define the topic and indicate the existing knowledge gaps. The verbiage should indicate both the specific nature of the topics well as the approach intended with the expressed purpose of generating the reader’s interest.

### Position statements

A position statement should be drafted based upon input from all contributing co-authors, following a comprehensive literature review and summary of current scientific evidence. A clear statement in point-form of the specific topic should be made, which cover the most important aspects of the topics with a focus on practical management.

Following the position statements, recommendations should be formulated based upon a grading system [7]. Every recommendation should be reviewed, and assigned a score based upon all co-authors and input from the WSES editorial board.

### Conclusion

The conclusion should be a brief summary of the paper and the position of WSES. If applicable, particularly important recommendations may be re-stated.

### Contributors listed in acknowledgments

All contributors who do not meet the criteria for authorship may be listed in an acknowledgments section at the conclusion of the manuscript.

## Development of PP

The idea for a PP may be initiated by any member of WSES and should be submitted to the Board of Directors, who can provide guidance, feedback, and potential collaborations which may benefit the process. The concept generally arises from major areas of practice or examines clinical issues where there is controversy or where there are multiple practices or therapeutic options.

There are different ways of producing PP. Either the WSES board or individual members with specific interest may lead to the appointment of the lead author and panel of experts/working group (WG). The WG may be defined as WSES members having expertise in specific areas related to the topic.

The WG then reviews and synthesizes the evidence, leading to well-designed power point presentations before all attendees at an annual WSES meeting. The presentations correspond to a critical aspect of the evolution of the manuscript, it provides an opportunity for the WG to meet, collaborate, and organize their ideas, as well as a chance for the WSES membership at large to provide valuable input and feedback to the group who are formulating the PP. The presentation should include background information, relevant abbreviated literature review which should assess the quality of the scientific evidence, and an evidence-to-recommendation table with specific and clear proposed practical recommendations. Based upon this session, the WSES leadership may accept or modify the proposed recommendations, or suggest potential improvements.

Following the presentation, discussion sessions should be moderated by one or more of the proposed co-authors, where various opinions and comments should be recorded for later reference. At the conclusion of the session, one of the proposed co-authors should conclude the session with a response to all raised comments.

Based upon the presentation at the WSES meeting and collaborated material feedback received, the WG can feel confident moving forward with drafting of the initial manuscript for publication. Of note, all co-authors should have access to the power point presentations as a guide to writing their respective sessions.

**Fig. 1:**
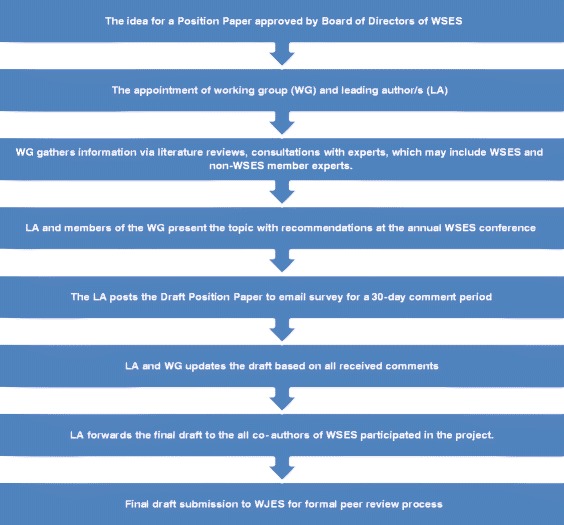
Steps for the development and approval of position papers on clinical practice by WSES

The lead author, with the Board’s approval, and selected experts (co-authors) generally prepare the first draft of the paper (Fig. 1). Once agreed upon by the first two or three authors, this draft may be shared via e-mail to all co-authors as well as the WSES board members for review and comment. The WG should only consider comments from respondents, who provide name, affiliation, and email.

The final draft is forwarded to all co-authors for an additional 2 weeks of review and final comments prior to submission to World Journal of Emergency Surgery (WJES).

Table 1 represents a flow diagram showing the complete process from inception to publication as described.

## Conclusion

A suggested algorithm for development of a position paper for WSES is presented, with the aim of creating a uniform process which will be user friendly for the membership in an effort to streamline the process. Furthermore, by adhering to such a process, we hope to create a valuable template for use in preparation for various information sessions at the WSES yearly meeting.

The appropriate and ethical production of position papers published by WSES will benefit the society and surgical community in general with the hopes of inspiring practice improvement and up to date clinical care.

## Abbreviations

PP: Position paper
WG: Working group
WJES: World Journal of Emergency Surgery
WSES: World Society of Emergency Surgery
